# Reproductive health and burn-out among female physicians: nationwide, representative study from Hungary

**DOI:** 10.1186/1472-6874-14-121

**Published:** 2014-10-02

**Authors:** Zsuzsa Győrffy, Diána Dweik, Edmond Girasek

**Affiliations:** Institute of Behavioural Sciences, Semmelweis University, Nagyvárad square 4, H-1089 Budapest, Hungary; Department of Obstetrics and Gynecology, University of Szeged, Semmelweis st. 1Szeged, H-6725 Szeged, Hungary; Health Services Management Training Centre, Semmelweis University, Kútvölgyi st 2, 1125 Budapest, Hungary

**Keywords:** Burnout, Female physicians, High-risk pregnancies, Miscarriage, Reproductive health

## Abstract

**Background:**

There is a worldwide rising tendency of women deciding to become physicians; hence, one of the most remarkable fields of investigation is the wellbeing of female doctors. The aim of this study was to describe female physicians’ reproductive health in Hungary and to explore the potential correlation between their reproductive disorders and burnout symptoms. Up to our present knowledge, there have not been any studies investigating the correlation between reproductive disorders and burnout of female physicians; therefore, our study represents a unique approach.

**Methods:**

Data in this representative cross-sectional epidemiological study were obtained from online questionnaires completed by 3039 female physicians. Participants in a representative nationwide survey (Hungarostudy, 2013) served as controls (n = 1069). Differences between physicians and the control group were disclosed by chi-square test. Correlations between certain factors of reproductive health and the three dimensions of burnout were detected by Pearson correlations and *X*^2^ test. Binary logistic regression analysis was used to determine the association between burnout and reproductive health.

**Results:**

Female physicians were more often characterised by time-to-pregnancy interval longer than one year (18.4% vs. 9.8%), were bearing more high-risk pregnancies (26.3% vs.16.3%), and were more likely to be undergoing infertility therapy (8.5% vs. 3.4%) and experiencing miscarriage (20.8% vs. 14.6%) during their reproductive years, compared with the general female population. With the exception of miscarriages, the difference remained significant in all comparisons with the professional control group. Both high-risk pregnancies and miscarriages of doctors were associated with depersonalisation (p = 0.028 and p = 0.012 respectively) and personal accomplishment (p = 0.016 and p = 0.008 respectively) dimensions of burnout. Results of the multivariate analysis showed that, beside traditional risk factors, depersonalisation acted as an important explanatory factor in case of high-risk pregnancies (OR = 1.086).

**Conclusions:**

There is a circulatory causality between burnout and the development of reproductive disorders. Burnout is an important risk factor for high-risk pregnancies and miscarriages, and it has a negative effect on the outcome of pregnancies. At the same time, women suffering from reproductive disorders are more likely to develop burnout syndrome. Improvement of working conditions and prevention of burnout in female doctors are equally important tasks.

## Background

### Reproductive health of female physicians

There is a worldwide rising tendency of women studying to become physicians. While forecasts in the 1990s prognosticated that every third medical doctor would be a woman by 2010, it has become clear by now that more than 50% of those studying and practicing medicine are women [1–4]. This gender realignment of the profession raises several questions, not only during university and postgraduate training, but also after specialisation. One of the most remarkable fields of investigation is the reproductive health of female doctors.

International papers give a heterogeneous view of the reproductive health of female physicians. According to Phelan’s study that involved 2,000 female doctors in the US, there was no difference between reproductive disorders of female doctors and those of the general female population [5]. Pinhas-Hamiel et al. found a higher rate of preterm births, but the same rate of miscarriages among Israeli house officers, compared with the general female population [6]. The metaanalysis of Finch became a cornerstone in the field of studying fertility disorders among female physicians, since it showed that health problems during pregnancy occurred more frequently among house officers and young female doctors [7]. A higher rate of health disorders during pregnancy was confirmed by two quantitative investigations [8, 9], and a higher rate of preterm births was found in a survey of Canadian female surgeons [8–10]. Hamilton et al. also found a higher rate of preterm deliveries among female surgeons, compared with the general female population [11]. However, a Finnish study (performed between 1996 and 2006) did not find a difference in health problems during pregnancy between the general population and female doctors [12].

Reproductive health of Hungarian female physicians has also been investigated since the new millennium. In the first qualitative study in 2002, a notable proportion of female doctors reported health problems that occurred during pregnancy and during the period between two pregnancies, compared with female pharmacists [13]. Another study, performed between 2003 and 2004, involved a representative sample of 408 female doctors and another sample of professional women, controlled for age and place of residency. In the comparison regarding reproductive health it was found that terminations of pregnancy, miscarriages and high-risk pregnancies were more frequent among physicians [14]. These findings were further supported by the results of another survey of female psychiatrists, who reported more terminations of pregnancy and miscarriages compared with the group of professional controls [15].

According to the results of our previous research, reproductive disorders are more frequent among Hungarian female physicians compared with the general population.

### Burnout syndrome among female doctors

Work stress is a remarkable risk factor that may affect pregnancy outcome [16, 17]. There is a trend that female doctors carry on working during pregnancy, even though their profession puts a significant physical and emotional burden on them.

Burnout can be seen as a special stress factor of the medical profession. The traditional definition of burnout was given by Freudenberger: “… a specific psychological condition in which people suffer emotional exhaustion, experience a lack of personal accomplishment, and tend to depersonalize others” [18].

This definition was complemented by Maslach and Jackson, who disclosed three main dimensions of burnout: emotional exhaustion, depersonalisation and reduced personal accomplishment [19].

Beside the abovementioned theoretical models, a new and complex conceptional framework of research into burnout has emerged in the international scientific field. Maslach et al. considered the lack of concordance between the individual and the work or working environment to be an important predictor of burnout [20]. There are six components of a working environment: workload; lack of control; insufficient reward and the accompanying feelings of continually having to do more for less; the feeling of community in which relationships become impersonal and teamwork is undermined; the absence of fairness; and conflicting values, in which choices that are made by management often conflict with their mission and core values. The lack of these components is the most important determinant of burnout syndrome [21, 22].

### Aims of the present study

We investigated the physical and mental status of Hungarian female physicians in a nation-wide representative survey in 2013. Investigating reproductive health and work-related background factors of reproductive health was one of our main objectives. According to our hypothesis and the results of our previous research, reproductive disorders are more frequent among Hungarian female physicians, compared with the general population. We also assume that reproductive health problems and burnout syndrome, which is a leading symptom of work stress, show significant correlation. Up to our present knowledge, there is no study that investigates the correlation between reproductive disorders of female physicians and burnout; therefore, our study represents a unique approach.

## Methods

### Study design and data collection

#### Online survey among female doctors

Our online quantitative survey took place between May 9 and July 15, 2013 and focused on physicians and dentists who worked in Hungary. A link to an anonymous online self-administered questionnaire was sent to all potential participants, followed by four reminder e-mails (sent every two weeks on average). Response rate increased continuously: 4000 physicians and dentists responded in the first fortnight of the research period, and a further 2607 responders were inspired by the reminder letters.

Among the population, we considered all those who were registered members of the Hungarian Medical Chamber and had a valid e-mail address (N = 42.342).

Response rate was 16.18% (n = 5.607), which is considered acceptable in comparison with the average response rate of online surveys [23].

Our data were weighted by gender, age and profession (physicians, dentists). After three-dimensional weighting, the data with regard to the region and type of workplace were compared with the same type of data from the Hungarian Central Statistical Offices. We considered our survey to be representative, because we did not find significant difference according to regions (by counties), nor according to type of workplace (general practice, inpatient and outpatient care).

The survey was performed with the permission of the Hungarian Medical Chamber. Ethical permission was obtained from the Ethical Committee of Semmelweis University, Budapest (No: 60/2013).

#### The cross-sectional representative study

The Hungarostudy 2013 survey, a large cross-sectional representative study, was conducted among the Hungarian adult population.

A clustered, stratified sampling procedure was used. From the 150 sub-regions of Hungary, all settlements with more than 10,000 inhabitants, as well as representative samples of smaller settlements, were included. Samples of 2,000 individuals each, which were representative according to gender, age and the sub-regions for the adult population of Hungary, were selected from the national Population Register. Structured interviews concerning demographic data, physical and mental health characteristics, psychosocial risk and protective factors and participants’ attitudes towards the health system were conducted [24].

### Measurement of reproductive health variables in both samples

The following five reproductive health factors were assessed:

1. Participants were asked whether they had had any miscarriages in their lives (Yes/No).
2. Participants were asked whether time-to-pregnancy had been longer than one year in case of any of their pregnancies.
3. Participants were asked whether any of their pregnancies were high risk (Yes/No).
4. Participants were asked whether they had ever been treated for infertility (Yes/No).
5. Participants were asked about how many terminations of pregnancy they had had in their lives (To be answered with a number).

In case of miscarriages, high risk pregnancies and terminations of pregnancy, the year of the last event was also asked.

### Ascertainment of covariates in both samples

The following age groups were applied: 24 to 35, 36 to 45, 46 to 55, 56 to 65 years, and older than 65 years. Marital status was coded into a two-category variable as ‘living with partner’ (married or cohabitating) or ‘single’ (single, living separately from partner, divorced, or widow) to increase statistical power. Answers to the item regarding the number of children were dichotomised into having or not having children, to obtain a proxy measure for parity. The variables of current workplace were categorised into four groups: working in inpatient care or outpatient care, working as a general practitioner or other (non-governmental organisation, private practice, etc.)

In the multi-adjusted binary regression analysis, we used the following variables: tobacco habits were categorised as ‘never smoked’ or ‘current’/‘former’ smoker. Self-reported height and weight were recorded, and body-mass index (BMI) was calculated. A question with regard to any ongoing gynaecological treatment and any induced abortions in history was also included. The variable regarding shift work was dichotomised.

### Assessment of burnout

Burnout was measured by the Hungarian version of Maslach Burnout Inventory-Human Services Survey (MBI) [19, 25]. The 22-item questionnaire is aimed to measure each of the three components of burnout (emotional exhaustion or depersonalisation, and lack of personal accomplishment) by three different subscales. Responses are rated on a 7-point Likert scale (0 meaning ‘never’ and 6 meaning ‘every day’).

The 22 items put up three subscales:

Emotional exhaustion subscale (EE) consists of nine questions and assesses the feelings of being overextended and exhausted in one’s workplace (e.g., “I feel depressed at work”).

Depersonalisation (DP) subscale has five questions and measures an unfeeling and impersonal response toward recipients of one’s service, care treatment, or instruction (e.g., “I don't really care what happens to some recipients”).

Personal accomplishment (PA) subscale consists of eight questions assessing feelings of competence and success in one’s work (e.g., “I have accomplished many worthwhile things in this job”) [19].

Using cut-off scores for the means based on data from a normative sample of 1104 health professionals in the United States, respondents are classified as high, moderate or low burnout cases on the respective subscales [19]. Among physicians, the cut-off points of high scores are as follows: EE: >27 score; DP: >10 score; and PA: <33 score. In our study, the Cronbach’s alpha coefficients for the subscales proved to be 0.909 for EE, 0.767 for DP and 0.818 for PA.

### Statistical analyses

Descriptive statistics were used to determine the prevalence of reproductive disorders among Hungarian female physicians. Characteristics of physicians and the control group were compared by χ2 tests. Correlations between certain factors of reproductive health and the three dimensions of burnout were detected by Pearson correlations and *X*^2^ test. All tests were 2-sided, with a type I error level of 0.05.

Unadjusted and multi-adjusted binary logistic models were performed to examine the association between burnout and the five reproductive health factors. In the multi-adjusted binary regression analyses, we adjusted for age, parity, tobacco habits, BMI, gynecological treatment, induced abortion, and shift work in the past few years. A threshold of *p* <0.05 was established in order to consider an association significant. Statistical analyses were conducted using the SPSS 15.0 for Windows.

### Ethical approval

The study was approved by the Ethics Comittee of the Semmelweis University, Budapest.(ref. number: 60/2013).

## Results

### Demographic characteristics of participants

#### Female physicians

The most important demographic characteristics of the respondents are shown in Table 1. All female physicians from the representative study, a total of 3039 subjects, were included in the present analysis. Medical doctors comprised 82.8% of the sample and the remaining 17.2% were dentists. The average age of the participants was 49.5 years. With regard to marital status, 10.1% were single, 13.5% cohabiting, 59.4% married, 11.3% divorced and 5.4% widowed. 23.8% were childless, while 21.2% of the respondents had one, 40.1% had two and 14.9% had three or more children. Distribution of the participants’ workplace was as follows: 35% worked in inpatient care, 24.6% in outpatient care, 26.1% in general practice and 14.3% somewhere else (e.g., non-governmental organisation, private sector).

**Table 1:** **Main socio-demographic characteristics of female physicians**

	Total	n = 3039
**Age (years)**	**%**	**n**
<35	21.5	646
36-45	19.1	574
46-55	22.3	672
56-65	22.8	688
>66	14.4	433
**Marital status**		
single	26.7	807
living with partner	73.3	2213
**Number of children**		
0	23.8	716
1	21.2	637
2	40.1	1206
3 or more	15.0	451
**Type of workplace**		
inpatient care	35.0	970
outpatient care	24.6	682
GP	26.1	723
others	14.3	396

One-third (33%) of female physicians worked in the capital, 28.7% worked in larger and 30.1% in smaller cities, whereas 6.3% worked in villages. In further analysis, three main categories were used: capital/larger city, smaller city and village.

Slightly more than 35% of the sample worked in inpatient care. Due to the heterogeneity of this group (e.g., night shifts), we examined them by medical specialties. Distribution of these respondents according to medical specialty is shown in Table 2.

**Table 2:** **Distribution of specialties among female physicians working in in-patient care**

Speciality	In patient care% (n = 818)
Anesthesiology	11.1 (91)
Dermatology	1.7 (14)
Emergency medicine	2.3(19)
Internal medicine	17.7 (145)
Neurology	5.8 (48)
Obstetics/gynecology	2.2 (18)
Ophtalmology	3.2 (26)
Otolaryngology	2.4 (20)
Pathology	2.4 (20)
Pediatrics	13 (106)
Preventive medicine	0.6 (5)
Psychiatry	7.1 (58)
Radiology	8.6 (70)
Rheumatology	4.2 (34)
Surgery	12.8 (23)
Others	7.8

#### The control group and the professional control group

A total of 1069 women (53.5%) participated in the nationwide representative survey, Hungarostudy 2013. The average age of respondents was 48.5 years. 17.7% were single, while 7.7% were cohabiting, 42.3% married, 12.4% divorced and 19.3% widowed. 25.8% were childless, whereas 21.7% had one, 33.8% had two and 18.7% had three or more children. The educational level of the respondents was as follows: 39.2% finished eight or less grades at elementary school, 14.3% were skilled workers, 32.2% graduated from high school, and 13.7% from college or university. The professional control group (women with a college or university degree) consisted of 146 women. The average age of professional women was 48.1 years. Almost one-fifth (18.1%) of the respondents were single, 11.1% were cohabiting, 48% were married, 18.1% were divorced and 7.8% were widowed. Almost one-third (31.9%) of them were childless, 29.3% had one, 33.8% had two and 4.9% had three or more children.

#### Socio-demographic correlates and different dimensions of burnout among female physicians

The mean score on the emotional exhaustion subscale of MBI was 19.23 (SD = 11.99), on the depersonalisation subscale 5.34 (SD = 0.54) and on the personal accomplishment subscale 34.60 (SD = 0.94). Levels of the different dimensions of burnout of female physicians are shown in Table 3. Analysing the different subscales according to age of respondents, we found that the medium and high scores reached, not only on depersonalisation but also on emotional exhaustion and personal accomplishment subscales, were characteristic of the youngest age group (Table 4).

**Table 3:** **MBI subscales among female physicians**

	EE ^*^% (n)	DP ^**^% (n)	PA ^***^% (n)
Low level	48.6 (1402)	62.3 (1782)	33.7 (968)
Moderate level	30.4 (876)	23.1 (662)	27.8 (798)
High level	21.0 (607)	14.6 (419)	38.5 (1106)

^*^EE: Emotional exhaustion.

^**^DP: Depersonalization.

^***^PA: Personal accomplishment.

**Table 4:** **Relation between MBI subscales and socio-demographic characteristics of female physicians**

		***EE*** ^*^	***EE***	***P***	***DP*** ^**^	***DP***	***P***	***PA*** ^***^	***PA***	***P***
		Low	Moderate and high		Low	Moderate and high		Low	Moderate and high	
Age	<35	42.8	57.2	<0.001	48.6	51.4	<0.001	22.8	77.2	<0.001
	36-45	41.8	58.2		57.3	42.7		33.6	68.4	
	46-55	44.6	55.4		64.1	35.9		36.5	63.5	
	56-65	50.2	49.8		69.3	30.7		38.7	61.3	
	>65	76.8	23.2		80.5	19.5		42.9	57.1	
Marital status	not partnered	50.8	49.2	0.173	61.2	38.8	0.480	33.0	67.0	<0.001
	living withpartner	47.8	52.2		62.7	37.3		33.9	66.1	
Number of children	none	42.0	58.0	<0.001	50.0	50.0	<0.001	24.4	75.6	<0.001
	1≤	50.5	49.5		66.3	33.7		36.7	66.3	
Physician type	physiscian	44.1	52.9	0.001	60.3	39.4	<0.001	33.5	66.5	0.685
	dentist	55.5	44.5		71.6	28.4		34.5	65.6	
Type of workplace	inpatient care	38.2	61.8	<0.001 0.000	52.7	47.3	<0.001	26.4	73.6	<0.001
	outpatient care	56.5	43.5		72.4	27.3		39.5	60.5	
	GP	48.5	51.5		61.5	38.5		37.8	62.2	
	others	56.1	43.9		67.9	32.1		37.0	63.0	
Workplace location	capital/county town	47.6	52,4	0.891	61.6	38.4	0.719	32.3	67.3	0.022
	town	48.5	51.5		63.2	36.8		37.3	62.3	
	village	47.3	52.7		61.3	38.7		40	60	

^*^EE: Emotional exhaustion.

^**^DP: Depersonalization.

^***^PA: Personal accomplishment.

There was no correlation between different levels of burnout and parity status (single or in a relationship). The medium and high levels of emotional exhaustion, depersonalisation and personal accomplishment were significantly higher among the childless physicians compared with those who had one or more children (Table 4). We explored the possible relationship between the workplace location and the different dimensions of burnout. Neither the level of emotional exhaustion nor that of depersonalization were associated with the type of the location. Furthermore, personal accomplishment showed very low levels in all three workplace locations (Table 4).

Emotional exhaustion and the depersonalisation were more remarkable among general practitioners than among dentists. Those who worked in inpatient care reached higher scores on all three subscales of MBI (Table 4).

According to the different specialisations of those having worked in inpatient care, no significant differences were detected in the emotional exhaustion and personal accomplishment levels. On the other hand, higher scores (medium and high level) of depersonalisation were shown among more than half of the dermatologists, rheumatologists and emergency physicians (data not shown). Our research has an important limitation: due to the wide range of specialties, there are only few women in each category; therefore, these results show only tendencies.

#### Indicators related to the reproduction of female physicians and the two control groups

Almost 80% of the respondent female doctors (n = 1905) had 1–8 pregnancies (mean = 3.06, SD = 1,57), and altogether 1836 children were born (mean = 2.5, SD = 1.2). At the time of the survey, two per cent of respondents were pregnant (n = 59).

Time-to-pregnancy was longer in 18.4% of the respondents (n = 538), and the pregnancy of 30.4% (n = 876) was imperiled by workplace hazards. The rate of those who were treated by infertility were 8.5% (n = 249), and among these women, 42.3% went through some form of artificial reproductive treatment. The association between the socio-demographic background and reproductive health of physicians is shown in Table 5.

**Table 5:** **Reproductive health disorders of female physicians in association with their socio-demographic characteristics**

		Time-to-pregnancy interval longer than one year	p	Infertility	p	High-risk pregnancy(ies)	p	Miscarriage (s)	p	Termination(s) of pregnancy	p
Age	<35	9.2	<0.001	7.4	<0.001	14.8	<0.001	9.0	0.318	4.4	<0.001
	36-45	29.3		13.3		32.7		24.0		12.9	
	46-55	21.1		9.8		29.4		24.3		28.2	
	56-65	17.8		6.6		29.2		24.2		29.4	
	>65	14.0		4.6		25.3		24.3		33.5	
Marital status	single	12.4	<0.001	5.5	<0.001	20.5	<0.001	16.5	0.001	24.0	0.016
	living with partner	20.5		9.6		28.3		22.4		19.8	
Number of children	no	14.4	0.001	9.5	0.342	4.3	<0.001	5.9	<0.001	7.9	<0.001
	yes	19.8		8.3		32.9		29.5		25.2	
Physician type	physician	18.8	0.679	8.8	0.482	27.5	0.001	21.7	0.008	20.7	0.243

High-risk pregnancy was reported by 26.2% (n = 763) of physicians, and 20.8% (n = 592) had had a miscarriage. Termination of pregnancy was mentioned by 21% (n = 603) of the respondents.

If we compare these data with the reproductive morbidity results of Hungarostudy 2013, there were fewer pregnancies in the general population, but the average number of children who were finally born was exactly the same (2.5). A higher prevalence of reproductive disorders among female doctors was shown by the higher rate of high-risk pregnancies, time-to-pregnancy longer than one year, infertility treatment and miscarriage among physicians. Prevalence of terminations of pregnancy was practically the same in the two groups of women. Comparing data of reproductive morbidity of physicians with the professional control group of Hungarostudy 2013, the same trends can be found, with the exception of miscarriages (Table 6).

**Table 6:** **Reproductive disorders among female physicians and the other two control groups**

	Female physicians	Control group	P values	Professional control group	P values
Time-to-pregnancy longer than one year	18.4	9.8	<0.001	15.8	0.008
Infertility	8.5	3.4	<0.001	5	0.005
Miscarriage(s)	20.8	14.6	0.023	18.2	0.266
Termination(s) of pregnancy	21.0	22.6	0.822	19.7	0.494
High risk pregnancy(ies)	26.3	16.3	0.001	18.2	<0.001

In the next step, we examined the correlation between reproductive disorders and burnout syndrome in female doctors. Since lifetime prevalence of reproductive disorders was queried, while burnout represents the past few years, we used a methodological restriction. Due to the retrospective character of the reproductive disorders, we took into consideration only those three disorders that showed up in the past few years: high-risk pregnancies, miscarriages and terminations of pregnancy. In the past five years (between 2008 and 2013), 7.6% (n = 146) of the pregnancies were high-risk pregnancies, five per cent (n = 97) of the respondents had had miscarriage, and 1.57% (n = 30) had had termination of pregnancy. Due to the low number of the cases, terminations of pregnancy were not examined further.

Following these steps, we looked for the correlation between the different components of burnout syndrome (Emotional Exhaustion, Depersonalisation, Personal Accomplishment) and high-risk pregnancies and miscarriages. During the analysis, both depersonalisation and personal accomplishment correlated significantly with the prevalence of high-risk pregnancies and miscarriages (Table 7).

**Table 7:** **The association between MBI subscales and certain reproductive disorders among female physicians**

	EE ^*^low%	EE mod/high%	p	DP ^**^low%	DP mod/high%	p	PA ^***^low%	PA mod/high%	p
High risk pregnancy(ies)	18.6	22.1	0.494	17.9	25.0	0.028	15.6	23.3	0.016
Miscarriage(s)	16.0	18.4	0.266	14.2	23.0	0.012	11.0	20.4	0.008

^*^EE: Emotional exhaustion.

^**^DP: Depersonalization.

^***^PA: Personal accomplishment.

We also performed multivariate analysis to unveil the association between the prevalence of high-risk pregnancies and miscarriages, and depersonalisation and decreased performance. In binary logistic regression analysis, we used binary variables of high-risk pregnancies and miscarriage as dependent variables, while independent variables were age, tobacco use, body mass index, the presence of gynaecological/obstetrical disorders, induced abortions and shift work (Tables 8 and 9).

**Table 8:** **The association between moderate and high level of depersonalization and certain reproductive disorders among female physicians**

Dependent variables	Unadjusted OR (95% CI)	Adjusted OR (95% CI)*
High risk pregnancy(ies)	1.532 (1.061-2.210)**	1.086 (0.761-1.671)
Miscarriage(s)	1.814 (1.154-2.852)**	NS*****

^*^ Adjusted for age, body-mass index, shift work, smoking and treatment due to gynecological disease and induced abortion.

^**^ p < 0.05.

^***^ p < 0.01.

^****^ p < 0.001.

^*****^ NS: Not significant.

**Table 9:** **The association between moderate and low levels of personal accomplishment and certain reproductive disorders among female physicians**

Dependent variables	Unadjusted OR (95% CI)	Adjusted OR (95% CI)*
High risk pregnancy(ies)	1.637 (1.096-2.444)**	NS^*****^
Miscarriage(s)	2.069 (1.217-3.519)***	NS*****

^*^ Adjusted for age, body-mass index, shift work, smoking and treatment due to gynecological disease and induced abortion.

^**^ p < 0.05.

^***^ p < 0.01.

^****^ p < 0.001.

^*****^ NS: Not significant.

After controlling to these variables, depersonalisation, beside other traditional risk factors, turned out to be an important explanatory variable in cases of high-risk pregnancies.

## Discussion

The reproductive health of Hungarian female medical doctors was mapped through a representative survey of doctors in 2013. Data were compared with the results of a representative Hungarian sample.

We found that compared with the general female population, a larger proportion of female medical doctors were characterised by time-to-pregnancy intervals longer than one year, and more of them had undergone infertility treatment or miscarriage(s). On the other hand, the same prevalence of terminations of pregnancy was found in both groups. Comparing data of reproductive morbidity of physicians and the professional control group of Hungarostudy 2013, the same trends can be found, with the exception of miscarriages.

Since we compared our data with those of the general female population, the negative trend of our data is more apparent: in general, educational level and health status correlate positively; therefore, one might expect highly educated women as less likely to suffer from reproductive problems [26]. We also assume that physicians are more familiar with methods of prevention and preservation of health, with options of early recognition and treatment of pathological conditions.

According to our original hypothesis, reproductive disorders and burnout, which is a crucial symptom of work stress, correlate with each other. Therefore, the first step of our study was to examine burnout among female physicians. Moderate and severe emotional burnout was found in cases of more than half of our respondents (51.4%). More than one-third (37.7%) of the participants reached moderate to high scores on the depersonalisation scale. Moderate or low levels of personal accomplishment were found in 66.3% of the cases. When examining socio-demographic characteristics of burnout, we found that high scores reached on all three subscales of burnout were significantly more frequent in cases of the youngest group of female medical doctors (24 to 35 years), in cases of childless women and among those who worked in in-patient care.

The next step was to explore the potential association between reproductive disorders and burnout. We applied methodological restriction to be able to precisely analyse the coincidence of reproductive disorders and burnout: only reproductive disorders (high-risk pregnancies and miscarriages) of the last five years were taken into account. It was found that high-risk pregnancies and miscarriages were in correlation with both depersonalisation and low personal accomplishment. Results of the multivariate analysis showed that, beside traditional risk factors, depersonalisation acted as an important explanatory factor in case of high-risk pregnancies. While data from the literature confirmed [27, 28], that of the three dimensions of burnout, the first one to show up was emotional burnout, our results showed that it only depersonalisation and low personal accomplishment were associated with high-risk pregnancies and miscarriages.

We found rather conflicting international results with regard to reproductive disorders of female medical doctors. On the other hand, according to our previous results, Hungarian female physicians seemed to be overrepresented compared with the general population, with respect to reproductive disorders [14]. We might assume the effect of more than just one factor when studying the background of reproductive disorders. Physical and emotional workload placed considerably heavy stress on young female doctors, who lack enough experience [28, 29]. The question of simultaneous harmonisation might also be an important issue. Furthermore, the harmonisation of different fields of life might be another difficulty: starting their professional lives and establishing a family simultaneously often leads to irresolvable conflicts [30, 31]. It is one of the Hungarian peculiarities that young female physicians are left almost unaided with the difficulties of starting their professional life. The effective mentor system, featured in international publications, is almost entirely missing in Hungary [32]. Another characteristic is the entire lack of part-time jobs for entrants in health care [14]. Furthermore, according to the results of a previous Hungarian survey, female medical students bear more altruistic, family-centred, idealistic ideas and show earlier commitment during their career socialisation process, compared with their male colleagues and female law students [33]. Another difference was highlighted by foreign studies reporting about the importance of ‘controllable lifestyle’: the role of the amount, characteristics and controllability of working hours is getting more and more relevant when launching one’s career [34]. On the contrary, Hungarian female doctors are basically driven by altruistic motivations, in spite of all the difficulties of their would-be profession, when choosing their medical profession and specialty [35].

Numerous surveys reported that changing shifts and the realignment of the circadian rhythm led to somatic disorders. It is possible that the heavy work load related to working in shifts, especially night shifts, has a significant effect on female reproductive functioning [36, 37]. International publications also emphasised that it was the younger generation of entrants (residents) who were most affected by burnout syndrome. [38].The first years of their professional life were characterised by enormous physical and emotional strain and very slight control over their work. Both factors have the potential to contribute significantly [39, 40].

Our survey highlights several new aspects contributing to research on burnout. While international surveys call attention to emotional exhaustion playing the key role, this dimension of burnout did not correlate with reproductive disorders in the case of our sample of female physicians. On the other hand, reduced personal accomplishment seems to be a more important factor, compared with the international trend. [41, 42] Since the importance of this dimension of burnout was emphasised by other Hungarians previously, it is possible that in the background there are special circumstances related to the local society and health politics [43]. According to certain domestic studies, the personal accomplishment dimension of burnout can be detected independently of the other two dimensions of burnout [44]. Nevertheless, background factors of this issue need to be studied further.

Burnout is strongly associated with heavy workload, as well as physical and emotional strain. It is also associated with stressful situations and one’s ability to cope with them [45]. The key factors in this process are emotional exhaustion, chronic fatigue and the depletion of cognitive stores. In our analysis, we handled the three dimensions of burnout as a certain indicator of complex work stress. We examined the potential association between burnout and reproductive disorders on the basis of this theoretical framework. Numerous studies revealed the link between burnout and physical and mental health, and many studies aimed to unveil the biological mechanisms in the background of burnout [45–50]. On the other hand, the correlation between burnout and reproductive health has only been examined in male samples; therefore, its analysis among women might supply a deficiency [51]. Based on the abovementioned results, we can assume that the effect of burnout on somatic and mental health extends to the field of reproductive health. However, the association between burnout and reproductive health can be explained by an approach in evolutionary psychology, namely when there are inadequate environmental conditions (such as more work stress and a greater workload), the adaptive response can be the postponement of pregnancy or reproductivity [52].

The most important factors that potentially jeopardise the reproductive health of female doctors are well documented in the literature: physical and chemical risks such as anaesthetic gas, X-ray, working in changing shifts and other psycho-social stress factors, such as long and unforeseeable working hours, and the lack of control over work or collegial support. A further important task is to create a balance between work and family, which can act as a significant source of stress among female physicians [53–55]. All the above mentioned factors mutually interact: work-related stress factors can directly and indirectly (by mediating burnout) influence reproductive health. On the other hand, incidental reproductive failure might result in compensatory hyperactivity in the workplace. This escape into work can lead to burnout in a one-track, uncompensated and abusive way.

One of the most important questions of our study pertains to the mechanisms in the background of the interaction between reproductive disorders and burnout. It is possible that burnout acts as a mediator in the development of reproductive disorders: we assume that in the background of burnout, the potential stress factors of the female medical profession can be found, such as emotional burden, urge to perform, and being pushed for time [56, 57]. It must be emphasised that it is not easy to differentiate between cause and effect in cases of burnout and its correlates. It is possible that there is a circulatory causality between burnout and the development of reproductive disorders. Burnout is an important risk factor of high-risk pregnancies and miscarriages, and it has a negative effect on the outcome of pregnancies. At the same time, reproductive disorders are more likely to cause burnout. This latter assumption was supported by the finding that there were more childless women who suffered from burnout in our survey. The balance between work and family plays a crucial role in the physical and mental health of female doctors, according to the international literature [58, 59]. These publications emphasise that reaching equilibrium is only possible when resources from different fields of life are mutually converted. According to our results, it is possible that healthy pregnancies and babies who are born play a substantial role in preserving the health of female physicians. Based on our survey, it seems likely that burnout is not a cause but rather a consequence of reproductive morbidity. Female doctors who face difficulties in the field of reproduction are potentially less successful in coping with burnout.

This study’s aim was to give a comprehensive overview of the association between Hungarian female physicians’ reproductive health and burnout. One of the strengths of our survey is that it was possible to compare our representative sample of medical doctors with a representative sample of the general population. Up to our present knowledge, there have not been any studies that surveyed burnout and reproductive disorders simultaneously, or compared the results with the data of a population-based survey. We used validated international tools to describe the dimensions of burnout. However, one of the limitations of our analysis was that although the association between the different variables was well-described, the cross-sectional character of the study prevented us from determining the causality precisely. Furthermore, it was not easy to compare our results with those of certain other international studies, since many of them used different methodology and tools. A further limitation of our study is that the group of female dentists was not examined separately. This was due to the low number of cases with reproductive disorders. Further research needs to explore the potential association between burnout and reproductive disorders among dentists.

Another limitation of our study was that a precise medical definition of high-risk pregnancy was not used; rather, it was based on self-report. At the same time, our survey raised very important questions in the field of physical and mental health of female physicians. Our results further stressed the crucial role of prevention: it is essential to help these women when beginning their careers and when establishing families by enhancing the mentor system, stress coping strategies, flexible working hours, etc. However, female doctors’ wellbeing is not the only field where prevention plays an essential role. Numerous publications affirm that a healer with burnout is not effective in his/her work. [60–62]. Psychic distress can lead to complications and malpractice, and, from a broader perspective, burnout in doctors can have a significant effect on the morbidity of the entire society. Therefore, physicians’ physical and mental conditions are important indicators of the functioning and effectiveness of the health care system [63, 64].

## Conclusion

Compared with the general female population, a larger proportion of female medical doctors was characterised by a time-to-pregnancy interval longer than one year, and more had undergone infertility treatment or miscarriage(s). It was found that high-risk pregnancies and miscarriages were in correlation with both depersonalisation and low personal accomplishment. Results of the multivariate analysis showed that, beside traditional risk factors, depersonalisation acted as an important explanatory factor in cases of high-risk pregnancies. Improvement of working conditions and the prevention of burnout are equally important tasks.

## Abbreviations

MBI: Maslach Burn-out Inventory
EE: Emotional Exhaustion
DP: Depersonalization
PA: Personal Accomplishment
SD: Standard Deviation
OR: Odd’s Ratio.
